# Selections and Abstracts

**Published:** 1916-06

**Authors:** 


### SELECTIONS AND ABSTRACTS

### HEALTH NEWS.

Issued by the United States Public Health Service
Release June 14, 1916.

Twenty-five out of every 1,000 employees in American industries, according to recent statistics, are constantly incapacitated by sickness, the average worker losing approximately nine days each year on this account. This "non-effective rate" for the great army of industrial workers in the United States barely suggests the total money loss to employers and employees. The lessened efficiency, the effects of reduced earnings in times of sickness, as well as the cost of medical attention, and the economic loss from deaths, swell the cost to industry and to the Nation to almost incalculable figures.

That much of this loss is nothing less than preventable waste and that this waste can be largely reduced by a properly conducted system of governmental health insurance for wage-workers are conclusions set forth in Public Health Bulletin No. 76, containing the results of a study of "Health Insurance—Its Relation to the Public Health," just issued by the United States Public Health Service.

The preventive value of health insurance is given especial emphasis in this study. "Any system of health insurance for the United States or any State should at its inception have prevention of sickness as one of its fundamental purposes," says the bulletin. "This country should profit by the experience of European countries where prevention is being recognized as the central idea necessary to health insurance if health insurance is to attain its greatest success in improving the health and efficiency of the industrial population."

Such a system, it is pointed out in the bulletin, would

1. Provide cash benefits and medical service for all wage-earners in times of sickness at much less cost than is now possible. Adequate medical relief would thus be placed within the reach of even the lowest paid workers who are most subject to ill-health.

2. Distribute the cost among employers, employees, and the public as the groups responsible for disease causing conditions and afford these groups a definite financial incentive for removing these conditions. This can be done by means of small weekly payments from employees, supplemented by proportionate contributions from employers and government at a rate reducible in proportion to the reduction of sickness.

3. Become an effective health measure by linking the co-operative efforts of the three responsible groups with the work of National, State and local health agencies, and by utilizing these agencies in the administration of the health insurance system.

4. Afford a better basis for the co-operation of the medical profession with public health agencies.

5. Eliminate the elements of paternalism and charity-giving by making employees and the public, as well as employers, joint agents in the control of this fund.

"A governmental system of health insurance," concludes the study, "can be adapted to American conditions, and when adapted will prove to be a health measure of extraordinary value."

---

### AN APPEAL TO THE AMERICAN PEOPLE.

With the calling of many thousand men into military service, new and heavy responsibilities fall upon the Red Cross, in its relation to the army and navy, on the one hand, and to the people of the United States on the other. At present these responsibilities are, first, to provide assistance to the medical services of the armed forces of the Government, by the organization of base hospitals, ambulance columns and other units for the care of our sick and wounded; second, to purchase, collect, forward and distribute supplies for our soldiers in the field, camp and hospital, to help soldiers' families left destitute and not provided for by other agencies.

For the necessary means to discharge this patriotic service, the Red Cross, in accordance with its custom, turns to the public, whose sympathy and generosity it has learned implicitly to trust. Upon the response to this appeal must depend the adequacy with which the Red Cross will be enabled to help our sick and wounded soldiers, to soften the harshness of field service, and to meet urgent needs among dependents at home.

In anticipation of such a grave emergency as this, the Red Cross has been building up an organization competent to meet the obligations of its National Charter and to make effective the generosity of a patriotic people.

Contributions may be sent to the treasurer of a local Chapter, or checks may be made payable to the American Red Cross and sent to national headquarters at Washington.

Wm. H. Taft,
Chairman, Central Committee,
American Red Cross.

---

The following statement has been issued by Major General Arthur Murray, U. S. A., Acting Chairman of the Central Committee of the American Red Cross:

Supplementary instructions for the collecting of military relief supplies as a result of the concentration of American troops on our Mexican border, just issued by the American Red Cross to its chapters and auxiliaries, name six intermediate depots in railroad centers of the United States where supplies should be sent by them to be assorted and classified, and three distributing depots from which final distribution of supplies will be made to the troops at the front. With two exceptions, Cincinnati and Denver, the exact addresses of the intermediate depots are given below. The points selected and didstricts embraced are as follows:

NEW YORK DISTRICT, Red Cross Supply Depot, Bush Terminal No. 19, 39th Street and Second Avenue, South Brooklyn, N. Y.: includes all New England and the Eastern part of New York State.

CINCINNATI DISTRICT, headquarters in Cincinnati, O.; includes Pennsylvania, the Western part of New York tained on application to chapters.

State, New Jersey, Delaware, Maryland, West Virginia, Ohio and Indiana.

CHICAGO DISTRICT headquarters Red Cross Supply Depot, Clearing Argo District, Chicago, Ill.; includes Minnesota, Wisconsin, Michigan, and Northern Illinois.

KANSAS CITY DISTRICT, Red Cross Supply Depot, care Montgomery, Ward & Co., Kansas City, Mo.; includes North and South Dakota, Nebraska, Iowa, Northern Missouri, and Northeastern Kansas.

DENVER DISTRICT, headquarters in Denver, Colo.; includes Montana, Idaho, Wyoming, Utah, and Northern Colorado.

SAN FRANCISCO DISTRICT, headquarters Red Cross Supply Depot, care Mr. A. B. C. Dohrmann, San Francisco, Cal.; includes Washington, Oregon, Nevada, and Northern California.

The headquarters of the following additional three districts are designated "distributing depots."

DOUGLAS, ARIZ., headquarters Red Cross Supply Depot, care Mayor W. H. Adamson; for all of Arizona and the Southern part of California (Los Angeles).

EL PASO, TEX., Red Cross Supply Depot, 516 San Francisco Street, El Paso, Tex.: for all supplies from intermediate depots at Kansas City, Denver, San Francisco and from all territory west of Kansas not otherwise embraced.

SAN ANTONIO, TEX., DISTRICT, headquarters Red Cross Supply Depot, Avenue E. and Fourth Street, San Antonio, Tex.; for all supplies from intermediate depots at New York, Cincinnati, Chicago, and from all southern states situated to the east of a north and south line running through the western boundary of Kansas.

Each depot will be under the charge of a manager who will have general supervision over the receipt, storage, and shipment of the supplies, and, in the case of distributing depots, of their final disposition. Instructions for the shipment of supplies to, and operation of, these depots have been prescribed at length—those relating to shipment of supplies may be ob-

These intermediate and distributing depots having been established by the Red Cross for the purpose of facilitating the distribution of supplies from all parts of the country to our soldiers and sailors, the Red Cross would be pleased to have all patriotic American citizens and associations desiring to give aid and comfort to them make use of the facilities thus afforded by its system of depots for the shipment and distribution of supplies on the Mexican border.

ARTHUR MURRAY,
Major General, U. S. A.,
Acting Chairman, Central Committee,
American Red Cross.

---

### THE NATIONAL BOARD OF MEDICAL EXAMINERS OF THE UNITED STAES

The need of a standard medical examining body for the whole United States and its Territories (tributary thereto) has occasioned the organization of The National Board of Medical Examiners. It is a voluntary board, the members of which are selected from the Medical Corps of the Army, the Navy, and the Public Health Service, the Federation of State Examining Boards, and other representative organizations, and the medical profession of the United States.

The aim of this Board is to establish a standard and certification of graduates in medicine, through which by the cooperation of the individual Boards of Medical Examiners, the recipients of the certificates of the National Board of Medical Examiners may be recognized for licensure to practice medicine.

The policy of the Board is to conduct its examinations on a broad scientific basis of such a high yet practicable standard that the holders of its certificates will receive universal recognition.

The independent action by the Board is furthered by the financial and moral support of the Carnegie Foundation.

The original Board consisted of fifteen members, as follows, and remains unchanged, except for the loss of the founder and secretary Dr. Rodman, who died on March 8, 1916. At a meeting June 13, 1916, Dr. W. L. Bierring, of Des Moines, Iowa, was elected to the Board.

Surgeon-General W. C. Braised, U. S. N., President; Dr. W. L. Rodman, Secretary; Colonel Louis A. LaGarde, U. S. A., Ret., Treasurer; Surgeon-General W. C. Gorgas, U. S. A.; Surgeon-General Rupert Blue, U. S. P. H. S.; Medical Director E. R. Stitt, U. S. N.; Assistant Surgeon-General W. C. Rucker, U. S. P. H. S.;Dr. Herbert Harlan, Federation of State Medical Examining Boards; Dr. Isadore Dyer, New Orleans, La.; Dr. Victor C. Vaughan, Ann Harbor, Mich.; Dr. Henry Sewall, Denver, Col.; Dr. Louis B. Wilson, Rochester, Minn.; Dr. E. Wyllys Andrews, Chicago, Ill.; Dr. Horace D. Arnold, Boston, Mass.; Dr. Austin Flint, New York, N. Y.

The permanent organization of the Board will consist of the three Surgeon-Generals and one other representative from each of the Government Medical Services, three representatives of the Federation of State Medical Examining Boards, and six members chosen at large from the medical profession by the National Board of Medical Examiners.

The official domicile of the Board is Washington, District of Columbia.

### Requirements.

### Requirements for Admission to the Examination.

Satisfactory completion of

(a) High School. A four-year high school course.

(b) College. Two years of acceptable college work, including physics, chemistry, biology, and a modern language.

(c) Medical School. Graduation from a Class "A" medical school. (American Medical Association classification.)

(d) Hospital Training. One year as interne in an acceptable hospital or laboratory.

The above requirements apply to graduates of medical schools in 1912 and thereafter. The Board may accept equivalent credentials in the case of graduates previous to 1912.

EXAMINATIONS.

The Board has been given spacious rooms in the Army Medical Museum for conducting its examinations. They will be conducted primarily by members of the Board, and will be written, oral, and practical, including the examination of cases. In addition to the written examinations held in the Army Medical Museum, oral, written, and laboratory examinations will be held also in the Army and Navy Medical Schools, and in the Hygienic Laboratory of the Public Health Services, these facilities, as well as the Government Hospitals wherein will be held clinical examinations, having been placed at the disposal of the Board for the purpose.

Credentials must be presented to the Board sufficiently early for investigation. If adequate time is not allowed for this purpose, credentials may be rejected.

The following subjects will be included:

1. Anatomy: Microscopic— Embryology, Histology and Organology, Neurology; Gross—Osteology, Dissection; Applied—Regional, Topographical, Surgical.

2. Physiology:

3. Chemistry and Physics: Organic, Physiological, Physics.

4. Pathology and Bacteriology: Bacteriology, Microscopic Pathology, Gross Pathology, Surgical Pathology.

5. Materia Medica, Pharmacology and Therapeutics: Materia Medica, Pharmacology, Therapeutics and Prescription Writing, Electrotherapeutics, including Radiotherapy.

6. Medicine: Theory and Practice, Physical Diagnosis, Laboratory Diagnosis, Diseases of Nervous System, including Psychiatry, Diseases of Children, Tropical Medicine.

7. Surgery: General, including Minor Surgery, Operative Surgery, Special Surgery—Ear, Nose and Throat, Eye, Genito-urinary, Orthopedics, Radiology, Skin Diseases, Syphilis and Venereal Diseases.

8. Obstetrics and Gynecology.

9. Hygiene and Sanitation: Sanitary Science, Epidemiology, Vital Statistics, State Medicine.

10. Medical Jurisprudence.

| | | |
|---|---|---|
| 1. | Anatomy | 100 |
| 2. | Physiology | 75 |
| 3. | Chemistry and Physics | 75 |
| 4. | Pathology and Bacteriology | 100 |
| 5. | Materia Medica, Pharmacology, and Therapeutics | 75 |
| 6. | Medicine | 200 |
| 7. | Surgery | 200 |
| 8. | Obstetrics and Gynecology | 100 |
| 9. | Hygiene and Sanitation | 50 |
| 10. | Medical Jurisprudenee | 25 |
| | Total | 1000 |

Passing grade is an average of 75 per cent.

A candidate receiving a mark below 50 per cent. in one subject or below 65 per cent. in two subjects, fails.

Candidates failing at the first examination may register for a second examination at the end of one year. A third examination will not be allowed.

It is expected that the examination will cover about one week.

No fee is charged for the examination itself, but a registration fee of Five Dollars will be required.

The first examination will be held in Washington, beginning October 16, 1916.

CERTIFICATION.

Candidates who have been successful in passing the examination and are approved by the Board, will be granted certificates.

This certificate is not a license to practice medicine, nor does it exempt the holders thereof from complying with the legal requirements of the States in which they desire to practice; but it will be evidence of high attainment in medical knowledge; and will, the Board believes, soon be accepted by State Boards as evidence of qualification for licensure.

Resolutions endorsing the National Board of Medical Examiners have been passed by the following:

The American Medical Association, The Council on Medical Education of the American Medical Association, The American Association of Military Surgeons, The American Roentgenological Association, Southwestern Medical Association, Mississippi Valley Medical Association, Southern Medical Association, Clinical Congress of Surgeons of North America, Western Surgical Association, St. Louis Medical Association, Milwaukee Surgical Association, Seaboard Medical Association, Harrisburg Academy of Medicine, etc.; Southern Surgical and Gynecological Association, Southern Medical Association.

Further information and application blanks may be obtained from the Secretary, Dr. J. S. Rodman, 2106 Walnut Street, Philadelphia, Pa.

---

### INEFFECTIVE APPENDICECTOMIES.*

By Robert T. Morris, M.D., New York.
Professor of Surgery, Post-Graduate School of Medicine.

So many ineffective appendicectomies are done upon a basis of incomplete diagnosis, that some of us must call a halt. The matter is getting to be a scandal. During the year a number of cases are sent in to my service in the hospital, with a wrong diagnosis of appendicitis. Some of these are believed to be cases of acute infection of the appendix, but a larger number are sent in with a statement that the appendix has given trouble for a considerable time and requires removal. I have not kept notes relating to the proportion of these cases which are not really ones of appendicitis, but it corresponds pretty closely with reports of what I hear of patients operated upon without result, so far as appendix symptoms are concerned.

Let us first consider a group of cases in which the appendix is really a factor in the patient's discomfort, but not the principal one. The two chief irritative lesions of the appendix, not infective, are fibroid degeneration and syncongestive appendicitis.

Fibroid degeneration seems to occur as a normal involution process, but its symptoms appear to be most marked in that group of patients who present a number of stigmata of arrested development. In neurasthenic patients with relaxation of peritoneal supports and in the patient with a defective chromaffin system, allowing various abdominal manifestations to appear, fibroid degeneration of the appendix adds a factor. Removal of the fibroid appendix leaves these patients with all of their other defects still present. Attention which has been concentrated upon symptoms in the appendix region, is then transferred to some other organ or structure, and the mental prodding which is given to symptoms gradually increases the symptoms to the point where the patient is quite as ill as before. Fibroid degeneration, however, is a definite pathological entity and sometimes an important precipitating factor for symptoms. The reason appears to depend upon irritation of terminal nerve filaments which remain in contracting hyperplastic connective tissue up to the time when practically all other structures of the appendix have disappeared.

An impulse from irritated nerve filaments is sent to the autonomic centres, to sympathetic centres in general, and to cerebrospinal centres. A number of reciprocal reactions then appear.

In cases of syncogestive appendicitis (the other common irritative lesion of the appendix) we have a distention of the inner coats of the appendix with interstitial infiltrates, along with similar infiltration of other organs beside the appendi ., because of some organic disease, with obstruction to the blood and lymph circulatory systems. The reason why the appendix speaks up is probably because the inner soft coats cannot swell freely within the tight outer sheath of the peritoneum, and an irritation of nerve filaments results in these appendixes. It follows that in these two irritative lesions of the appendix, removal of that organ simply disposes of one unimportant factor in the patient's symptoms, and the cases as a whole must be taken into consideration or the operation will be a failure.

A still larger group of cases in which the appendix is removed ineffectively includes patients who never had appendicitis at all. Concerning the acute cases I have commonly found that the symptoms were due to some infective lesion of the pelvis or some infective lesion of the stomach or gallbladder, with accompanying tenderness of groups of abdominal sympathetic ganglia. Sometimes the cases have been bubonocele with pinching of a small area of bowel; sometimes they have been cases of pneumonia with pain referred to the right lower abdomen, and sometimes they have been cases of toxic irritation of spinal cord centres with efferent impulses sent out along the ilioinguinal and iliohypogastric nerves. The reason why these toxic neuroses appear oftenest on the right side is perhaps the fact that the ascending colon is such a fertile focus for enteric toxemia. Not only are patients of this group sent in frequently for operation at the hospital, but I am sometimes called to a distance and asked to be prepared for immediate operation upon the appendix.

A still larger group of cases with symptoms in the appendix region, but not including the appendix, appears to depend upon a large and complicated variety of irritative lesions of the spinal cord, with efferent impulses sent out, not only to the cerebrospinal nerves, but also to the sympathetic nervous system, and possibly to the autonomic centres, although Langley and his followers appear to believe that the autonomic system does not send out efferent impulses.

The most important single diagnostic point upon which I depend is one to which I called attention some years ago, and which is now included in a number of textbooks. If we press about an inch and a half to the right of the navel and a trifle below, and if we press deeply enough to bring out a response from the right group of lumbar sympathetic ganglia, there is definite evidence that we are to look to the appendix for the source of that particular irritation, provided that the left group of sympathetic lumbar ganglia is not hyperesthetic. This point of tenderness situated several inches away from McBurney's point is one which relates to a chronic irritative lesion of the appendix. Having determined that the appendix is a seat of chronic disturbance, we are still left to exercise surgical judgment, and to determine if removal of this factor of disturbance will decapitate the demon of all of the patient's ills. This means that we are to determine if the patient is one with neurasthenic habits and relaxation of peritoneal supports; if the patient is one with chronic enteric toxemia; or if the patient is one with syncongestive appendicitis along with important organic lesions at a distance.

If the right group of sympathetic lumbar ganglia is not hypersensitive on pressure we may practically rule out the appendix altogether. This may be done in perhaps the larger number of cases which are sent in with the diagnosis of chronic appendicitis, but in which symptoms are dependent upon impulses from various cerebrospinal or sympathetic centres.—New York Medical Journal, May 20, '16.

---

### CHRONIC CONSTIPATION IN THE AGED.

By I. L. Nascher, M.D., New York City.

In speaking of the aged I refer to the physical condition, not to the years of life. Of four hundred consecutive cases of aged persons who gave me a history of constipation, thirty were under fifty years of age. There were 150 men and 250 women, but about 100 women were seen in the female internal medicine department of a dispensary, the proportion in private practice being about equal. My object primarily was to find out to what factors they themselves ascribed their constipation; but as most of them said they were constipated as long as they could remember, this line of questioning was given up. Many were so accustomed to this condition that they did not consider it pathological and many paid so little attention to the evacuations that they could not recall when they had a movement of the bowels or even when they urinated last. It was, however, possible to bring out some facts bearing upon the etiology of chronic constipation in this class.

### Anatomical Changes.

Let us consider for a moment the anatomical changes in the process of senile involution which influence the formation, propulsion and evacuation of the feces. These are: diminished gastric juice, bile, pancreatic juice, mucus and the secretions from the intestinal glands, due to atrophy of these glands; atrophy of the muscular fibres in the intestines; formation of colonic and rectal pouches due to dilatation of the gut; enteroptosis; dilatation of the hemorrhoidal veins; flaccid abdominal walls, and probably some change in the nerve terminals. There are in consequence of these changes, lessened digestion and assimilation, with consequent lessened appetite; less food is taken and there is less waste; peristalsis is slower and less powerful, consequently propulsion is slower; the feces become dryer and travel along with more difficulty; they become lodged in the rectal or colonic pouch; there is diminished desire for food; there is dyschezia or difficult expulsion due partly to weakness, partly to the flaccid abdominal walls which the aged individual is unable to bring to his assistance in the act of expulsion, and partly to the hard feces. If there are hemorrhoids the difficulty is increased.

I am not considering in this paper such exceptional causes for chronic constipation as bands, growths and other pathological conditions which compress or obstruct the bowel. In aged persons the fecal mass travels so slowly through the bowels that it may take from 48 to 72 hours from the time of making a meal before the food fragments or waste is voided. Where there is but little waste to cause peristalsis, it may remain in the rectal pouch for two or even three days before there is a sufficient amount to create the necessary irritation to call for evacuation.

### Bad Bowel Habits.

If a person, accustomed to a daily stool, finds that with advancing age he must make a forcible effort to have a daily stool and the stool is then small, he will be inclined to wait until he has a strong desire for stool and thus he may acquire the habit of having a normal stool every second or third day. If he has a normal movement every second day without assistance from cathartics or enemas, it is not constipation and nothing should be done to secure a daily stool.

Many aged persons do not have a natural movement at regular intervals, but must either resort to cathartics or else must make forcible efforts to have a stool when they feel uncomfortable or have a headache or other symptom that they ascribe to constipation.

Many aged persons, and younger ones, too, pay no attention to this provision of nature to rid the system of waste but go only when they feel distressed. Some dread the act of defecation owing to the pain produced by the passage of hard feces or because they have piles; others dread the cold toilet in winter and thus they begin to neglect the call for stool until they can no longer hold back. In many cases the call for stool is slight and if not immediately attended to the desire passes away until a much larger amount of fecal matter in the rectum creates a greater irritation and the consequent pressure symptom which constitutes the call for evacuation. Some persons have so accustomed themselves to cathartics that evacuation will not occur without this extra stimulation. Lack of exercise is a frequent cause for the chronic constipation of the aged. There is then little waste, little appetite, little food is taken, and the small amount of fecal matter remains for days in the rectal pouch. In some cases there is impaired mentality; the patient ignores the desire for stool and the subconscious control over the sphincter is diminished, there being, at times, constipation, diarrhea or dribbling of stool. In these cases the underwear is usually soiled. (This conscious soiling of the underwear where the sphincter muscle is not impaired is a clear evidence of weakened mentality.)

In the dispensary referred to, the condition of the bowels is a routine question in taking the history. Over 80 per cent. give a history of constipation, although very few mention it as a symptom of their complaint; over fifty per cent. of their ailments are due to constipation, yet very few come to be treated for this condition.

### Symptoms.

The symptoms of chronic constipation can be classed in two groups; gastro-intestinal symptoms due to the presence of feces in the intestines, and toxemia symptoms due to the absorption of fecal matter and the products of intestinal decomposition.

Lane (British Medical Journal, November 1, 1913), mentions seventeen symptoms and pathological conditions due to intestinal autointoxication following intestinal stasis. In a paper on "Lane's Autointoxication Complex and the Manifestations of Senility" (New York Medical Journal, August 8, 1914), I pointed out that most of the symptoms mentioned by Lane were normal physiological manifestations of senility and others were pathological conditions to which the aged were peculiarly liable. Some of the latter may be due to autointoxication, but it requires more faith than I possess to accept this as the cause of such conditions as cancer, prolapsed uterus and grey hair.

The only symptom frequently met with in the aged which can be ascribed to autointoxication is a dull headache which makes the individual apathetic or irritable. There are many other symptoms which the aged individual suffering from chronic constipation may present and which may be due to autointoxication, but I am not satisfied to ascribe to this every symptom for which I can find no direct cause. It may sometimes be necessary to use this makeshift of an explanation to satisfy a skeptical patient just as we make a diagnosis of "old age" when we do not know what ails the aged patient.

The gastro-intestinal symptoms which the aged patients suffering from chronic constipation usually present are: discomfort in the abdomen and a feeling of fulness, weight or pressure in the pelvis; occasionally cramps, more often a dull ache or rather a sense of discomfort. There are sometimes waterbrash, eructations of gas and flatulence. The appetite is generally poor, tongue coated and breath foul. In rare cases the intestinal distention is so great as to press upward upon the diaphragm and interfere with the heart action. Upon examination the abdomen is found distended and tympanitic, the tympanites being marked or confined to the colon, the ascending colon being usually more distended and tympanitic than the other parts. Where there are thin abdominal walls the distended gut may stand out like a sausage and it is sometimes possible to see the bend at the hepatic flexure. In some cases light palpation with the finger tip may reveal a depression at the inner angle of the hepatic flexure showing a kink in the gut. Occasionally fecal matter can be felt in the gut, generally in the lower part of the descending colon. In many senile cases the rectal pouch is constantly filled with a mass of feces and sometimes this mass is so hard that it must be scooped out.

The stomach is usually distended and ptosis of the organ is found in cost cases. This can sometimes be determined by inspection, sometimes by light palpation.

STATEMENTS OF THE AGED UNRELIABLE.

The statements of the aged concerning pain and tenderness are unreliable, for they are always looking for sympathy and will exaggerate or lie, declare that any spot to which their attention is directed is painful, although a moment later, if their attention is directed to another spot or subject, considerable pressure can be applied to the former spot and they will evince no sign of pain. Their facial expression is a fairly reliable guide to their sensations. Pressure upon the distended colon may cause momentary spasm or colicky pain but if the pressure is applied in the direction of the fecal stream, upward along the ascending colon, from right to left along the transverse colon and downward along the descending colon, and the patient is cautioned not to restrain the inclination to pass flatus, pressure will give relief.

Accidents may happen when the old man lets up on the instinctive control over the sphincter and if more than flatus passes the sympathetic physician will console the patient instead of chiding him. In some cases there will be a constant sense of discomfort in the pelvis, a persistent desire to have stool but straining brings only small, hard, dry balls, lumps or flakes of feces and no relief from the discomfort. In these cases there is in the rectal pouch a mass of hardened feces from which flakes or lumps are broken off or else the mass is grooved and tunneled and feces lodged behind the mass are forced through in lumps which become rounded while passing through the groove or canal. Dyschezia or difficulty in passing stool may be due to lack of power to exert the necessary force to expel the feces. This occurs in very old persons who have flaccid abdominal walls.

### Chronic Cases.

As the chronic constipation of the aged is generally due to senile atrophy of muscular fibers and it is impossible to repair this waste, a cure of cases in which such atrophy exists is impossible. Much can, however, be done to cause regular stools and avoid the pernicious side effects and after-effects of chronic constipation. Many physicians treat senile constipation without giving the slightest thought to the underlying causes; many do not consider the effect of the various forms of cathartics. They seem to know no more about this than the layman who uses salines, enemata, peristaltic stimulants, massage, cholagogues, etc., indiscriminately, basing choice upon taste or convenience of administration.

### Treatment.

Before deciding upon the method or measures for treatment let the physician consider the basic cause or causes of the constipation. Is it diminished tonicity and lessened peristalsis due to muscular waste, or is there a deficiency in gastric or intestinal juices, bile, pancreatic juice or mucus, or is there an enteroptosis kinks, dilated rectal pouch, flaccid abdominal walls and general weakness, or are there several of these conditions present or is it a condition brought on by habit and neglect without anatomical changes? Each requires individual treatment. At the present moment mineral oil is the fad and physicians use it for all forms of constipation.

### Mineral Oil.

Mineral oil is in no sense a cathartic, its action being simply a lubricant, taking the place of mucus in the bowels. I recommended it for this purpose several years ago. (American Practitioner, August, 1913.)

The employment of oil in constipation is very old. In Floyer's "Medicina Geronomica," published in London, in 1724, the first scientific work dealing with the diseases of the aged, there appears this passage: "Oyl moistens the Excrements and skins of the Guts in old Men, who are subject to Costiveness; the peristaltic Motion is decayed, and wants a Stimulus also."

It is of special value in the aged, as the atrophy of the mucous glands with the consequent diminution of the secretion is one of the most frequent causes of slowed propulsion and prolonged retention of feces. It should be remembered that the fecal mass is fluid in the small intestines and it does not begin to harden and become formed until it reaches the caecum. The action of the lubricant is therefore desirable in the large intestines. But when it is used in excess or for a long time it probably coats the small intestines and prevents the discharge of the intestinal secretions, thereby interfering with intestinal digestion. In several cases in which I gave mineral oil in tablespoonful doses three times a day for two weeks there was a like effect in all. There was a copious daily stool which was soft, oily and passed without difficulty. But the patients lost in weight and upon examining the stools they were found to contain a large amount of undigested food particles including meat fibres, grains of corn, oats, rice, bits of milk curd, etc., all covered with oil. Mineral oil will do good in some senile cases if it is used for a short time. But its mode of action should be understood and it should not be used constantly or where the constipation is due to intestinal atonicity alone. It is useless in cases where there is dyschezia due to weakness, where there is a dilated weakened rectal pouch in which the fecal mass may remain for days, or where there is constipation due to habit.

### Diminished Peristalsis Due to Atrophy.

As the most frequent cause of senile constipation is diminished peristalsis due to atrophy of the muscular fibres of the intestines it would seem that the appropriate treatment is to give peristaltic stimulants. But anything which increases the activity of a degenerating muscle hastens its degeneration. The normal degeneration of the muscular fibres is a slow process and the normal stimulation produced by the cellulose of food will not unduly hasten the degeneration. The regulation of the food should therefore be the first measure taken to increase the peristaltic activity. In most cases, however, this condition has existed so long and the system has become so accustomed to drug stimulation that forced peristaltic stimulation is necessary.

### Other Drugs.

The best drug for this purpose is aloes or aloin. This increases peristalsis from the stomach to the rectum, its action being most powerful in the lower bowel. The main objection to its use is its griping effect, this being probably due to the compression of the nerve terminals by the contraction of the muscular fibers which cause peristalsis. The usual method of overcoming the griping is by the addition of belladonna. Belladonna relieves the griping by inhibiting contraction of unstriped muscular fibers, thus counteracting the action of the aloin: in other words, the belladonna prevents the full effect of the aloin. Aloin and belladonna is an irrational combination. Physicians will get as good results with a much smaller dose of aloin, and if they will add rhubarb to the aloin instead of belladonna they will overcome the griping and add a stimulant to the intestinal glands.

### Peristaltic Stimulant.

In senile cases where I want to use a peristaltic stimulant I generally prescribe a pill containing 1-10th grain aloin and 5 grains rhubarb. This will produce a soft, copious stool with little or no griping. Cascara is a reliable peristaltic stimulant, producing but little griping pain, but it is much slower in action.

Senna is useless unless given in large amount. None of these drugs should be given daily except perhaps for a few days after beginning treatment.

If the stools are light colored and foul smelling there is a deficiency of bile. In senile cases where there is a deficiency of a secretion which can be supplied artificially this should be done in preference to the stimulation of the secreting organ. If the deficiency is a temporary condition which can be relieved by slight stimulation of the organ and the organ is not in process of degeneration there will be no harm in stimulating the organ, but deficiency of bile in the aged is usually due to hepatic fibrosis. It is far better in these cases to give the bile salts than to give calomel or other cholagogues.

### Saline Cathartics.

Saline cathartics should not be used by the aged, as they withdraw water from the organism which the body cannot spare. They may be of service in obese individuals, but the benefit which such persons derive at the mineral springs must be ascribed more to the strict regimen enforced at the springs than to the waters, for they do not get the same effects when taking the waters at home.

The enema is only of service in retention of feces in the lower bowel. In cases where there is prolonged retention of feces in the rectum and the feces come out in hard, dry lumps, flakes or balls, an enema containing a dram of bicarbonate of soda to the pint of hot water should be used every second d[illegible], and on alternate days a suppository containing a half grain of ergotin should be used to increase the tonicity of the bowel.

### Astringents.

Astringents may diminish the dilatation but they will not increase the peristaltic force of the gut. The suppository should be pushed up as far as possible. If the alkaline solution used in the enema will not soften the mass in the bowel it will be necessary to use a scoop to extract the hardened feces. We find occasionally decrepit individuals with thin flaccid abdominal walls, who are so weak that they must exert all their strength to expel the feces. In these cases a soft stool should be secured either through the use of mineral oil or rhubarb, the seat of the toilet should be lowered or a foot stool provided to secure a more favorable position for defecation, the patient should wear an abdominal binder and efforts should be made to strengthen the individual.

THE DIET.

The most important general measure in the treatment of chronic constipation in the aged is the regulation of the diet. Digestion and assimilation being weakened in the aged, and as there is less physical activity, the aged eat less than in earlier life; there is less waste, and a smaller amount of cellulose is consumed to furnished peristaltic stimulation, though greater stimulation is necessary on account of the lessened tonicity of the intestines. Much more food than the aged person could consume would be necessary to furnish the inert material in sufficient quantity to bring about the necessary peristaltic activity, if he were permitted to follow the ordinary dietary of the home. An ideal diet, based upon calories and taking into consideration the need for increased amount of cellulose, could be devised but in the ordinary treatment of these cases it is impracticable.

A few general directions will suffice for the usual run of cases as we meet them in practice. The "little and often" rule of feeding is wrong. Food should not be given oftened than in 5- or 6-hour intervals. Meat should be thoroughly boiled, and when the teeth have fallen out very little meat should be taken and this should be chopped fine. No cold storage, canned or preserved meat or fish should be used. Spinach, cabbage, beets, carrots, turnips, cauliflower and sweet potatoes contain much cellulose. Thorough boiling of all vegetable foods; no fried food. Rice and sago constipate; other cereals do not. Whole wheat bread, graham bread and rye bread are better than the bread made from the very white, bolted flour. All fresh bread tends to constipate. The much-advertised breakfast cereals and foods are all good, but one breakfast food which contains 25 per cent. of bran is especially good in these cases. Figs, prunes and stewed apples have a slight laxative effect, but hardly enough to be of service unless taken in large quantities. Of the ordinary beverages, cocoa and tea constipate, boiled milk is often constipating and raw milk constipates some persons, while it acts as a laxative to others.

For a short time after beginning the treatment of these cases the diet should consist partly of concentrated, preferably predigested food and partly of food containing much cellulose. If under this treatment the regulation of diet and the use of drugs, there is an improvement, the physician should restrict or diminish the amount of drugs and adhere to the strict diet. There should be no departure from this diet until there are regular stools without the use of cathartics and enemas.

WATER DRINKING.

Many old persons have a daily stool if they take a glass of hot water with or without a little salt before breakfast. These persons do not suffer from chronic constipation but from chronic gastric catarrh. The hot saline solution liquefies the mucus which has accumulated during the night and which adheres to the walls of the stomach. This mucus after being loosened from the gastric walls, passes into the intestines and aids in the propulsion of fecal matter through the bowels.

PHYSICAL MEASURES.

I have not touched upon massage and other physico-therapeutic measures, as I have had no experience with them. I have also omitted those cases of chronic constipation that are due to mechanical obstruction or compression of the gut and which require surgical intervention. These conditions are, however, rare in the aged, the only operative condition at all frequent being hernia. The feces are generally sufficiently soft in the ascending colon to pass any kink at the hepatic flexure that does not completely compress the gut, and kinks at other locations and bands do not appear to play an important part in the senile constipation. I have not seen a case in the aged where surgical interference for such a condition was indicated, although surgeons will sometimes suggest operation for what they diagnose as adventitious bands. The measures recommended in this paper usually suffice to relieve if they cannot cure the chronic constipation.—The Medical Council, Philadelphia, May, 1916.

